# Africa Centres for Disease Control and Prevention’s framework for antimicrobial resistance control in Africa

**DOI:** 10.4102/ajlm.v7i2.830

**Published:** 2018-12-06

**Authors:** Jay K. Varma, John Oppong-Otoo, Pascale Ondoa, Olga Perovic, Benjamin J. Park, Ramanan Laxminarayan, Rosanna W. Peeling, Constance Schultsz, Han Li, Chikwe Ihekweazu, Amadou A. Sall, Baboucarr Jaw, John N. Nkengasong

**Affiliations:** 1 Africa Centres for Disease Control and Prevention, Addis Ababa, Ethiopia; 2 African Union, Inter-African Bureau for Animal Resources, Nairobi, Kenya; 3 African Society for Laboratory Medicine, Addis Ababa, Ethiopia; 4 National Institute for Communicable Diseases, Johannesburg, South Africa; 5 University of Witwatersrand, Johannesburg, South Africa; 6 United States Centers for Disease Control and Prevention, Atlanta, Georgia, United States; 7 Center for Disease Dynamics, Economics and Policy, Washington, D.C., United States; 8 Princeton University, Princeton, New Jersey, United States; 9 London School of Tropical Medicine and Hygiene, London, England, United Kingdom; 10 Amsterdam Institute for Global Health and Development, Amsterdam, the Netherlands; 11 China Center for Disease Control and Prevention, Beijing, China; 12 China PLA Institute for Disease Control and Prevention, Beijing, China; 13 Nigeria Center for Disease Control, Abuja, Nigeria; 14 Institut Pasteur, Dakar, Senegal

Antimicrobial resistant (AMR) organisms are increasing globally, threatening to render existing treatments ineffective against many infectious diseases.^1,2^ AMR strains of bacteria, fungi, parasites, and viruses prolong illness, increase case fatality, facilitate transmission, and increase treatment costs.^3,4^ In Africa where many health systems are weak, the likelihood of AMR increasing and the consequences of AMR infections are particularly high, and drug resistance has already been documented for HIV and the pathogens that cause malaria, tuberculosis, typhoid, cholera, meningitis, gonorrhoea, and dysentery.^5^ Patients in these countries have limited access to accurate diagnosis and adequate antimicrobial treatment, which can lead to sepsis and other life-threatening complications.^6,7^

Many factors contribute to the emergence, persistence, and transmission of AMR. Although AMR strains arise naturally due to genetic changes in replicating microorganisms, their emergence is accelerated by inappropriate use of antimicrobial agents in humans, animals, and the environment.^8^ AMR emergence may be amplified by substandard or counterfeit antimicrobials, which impair treatment of existing infections and may select for AMR strains.^9^ Transmission of AMR is accelerated by inadequate infection prevention and control in health care facilities, by contamination of the food supply with AMR bacteria, by impaired access to potable water, and by limitations in public health prevention programmes, including immunisation, sanitation, and sexual health.^10^ Globally, drug resistance causes an estimated 700 000 deaths each year, and if current trends continue, AMR could result in over 10 million deaths per year and over $100 trillion (United States dollars) in lost output globally by 2050.^11^

Recognising the urgent need for action, the World Health Assembly adopted the Global Action Plan on Antimicrobial Resistance in May 2015, and the World Health Organization established the Global Antimicrobial Resistance Surveillance System.^12^ In 2016, African countries committed to address AMR as part of the United Nations General Assembly special meeting and, in June 2017, committed to accelerate implementation of the International Health Regulations and promote Africa’s health security, of which AMR surveillance and control is a core component.^13,14^ To implement these commitments, the Africa Centres for Disease Control and Prevention (Africa CDC) officially launched its ‘Framework for Antimicrobial Resistance Control, 2018–2023’ in October 2017.^15^ Established in January 2017, Africa CDC is a ‘specialised technical institution’ of the African Union (AU) responsible for strengthening capacity for surveillance and disease intelligence, emergency preparedness and response, laboratory systems, information systems, and public health research and workforce development in Africa.^16^

Africa CDC developed its framework with four principles in mind. Firstly, the strategy should help advance the Global Action Plan. Secondly, while more information is needed about incidence, prevalence, and interventions, Africa CDC should follow the ‘precautionary principle’ and take action against resistance now. Thirdly, policy and practice are as important as diagnostic or treatment technologies in mitigating AMR. Finally, Africa CDC has an opportunity to leverage its unique position in the AU to raise awareness, secure commitments, and influence policy at the head of state level. After consultation with subject matter experts in Africa and globally, Africa CDC developed a framework that has four objectives: (1) improve surveillance of AMR organisms among humans and animals, (2) delay emergence of AMR, (3) limit transmission of AMR and (4) mitigate harm among patients infected with AMR organisms. Table 1 lists the high priority activities Africa CDC will pursue for each of these areas.

**TABLE 1:** Priority areas for Africa Centres for Disease Control and Prevention framework for antimicrobial resistance control, 2018–2023.

Strategic plan area	Activity
Improve surveillance	Increase the number of tests performed on humans and animals for AMR organisms
	Increase the proportion of clinical diagnostic laboratories with quality assurance programmes
	Increase the proportion of public health laboratories with quality assurance programmes and international accreditation
	Increase the number of national public health laboratories conducting surveillance for AMR using standardised protocols
	Increase the number of member states that continuously collect, analyse, report, and disseminate data about AMR for high priority pathogens in their respective countries
Delay emergence	Increase the proportion of physicians adhering to prudent antibiotic use guidelines
	Increase the proportion of veterinarians and food producers adhering to prudent antibiotic use guidelines
	Reduce availability and sales of substandard and counterfeit antibiotics
Limit transmission	Increase the proportion of health care facilities implementing infection control and prevention programmes
	Increase the availability and sales of animal products raised with prudent antibiotic use
Mitigate harm	Increase the number of health care facilities with quality diagnostic tests for infection and AMR
	Reduce the availability and use of substandard diagnostic tests and supplies
	Increase the proportion of physicians and health care facilities adhering to guidelines for treatment of susceptible and AMR infections in humans
	Maintain access to essential antibiotics
Cross-cutting	Advocate for policies and laws to enable long-term prevention and control of AMR
	Civil society engagement
	Develop human resources for AMR surveillance and control

AMR, antimicrobial resistance.

## Improve surveillance

Understanding the full extent of AMR and its impact in Africa is challenged by a lack of continent-wide AMR surveillance data, especially for pathogens that require complex testing methods.^3^ Although gains have been made in collecting data on resistance to some pathogens, such as HIV, *Mycobacterium tuberculosis*, and *Plasmodium spp.*, several challenges remain, including inadequate demand by clinicians for diagnostic testing, laboratory infrastructure, resources to continuously collect, transport, and test specimens for AMR surveillance, use of standardised protocols, quality assurance, systematic surveillance of AMR in animals and their products in Africa, and collaboration between the human and animal health sectors.^17,18^ Africa CDC’s framework identified several areas in which it can strengthen laboratory capacity for surveillance in humans and animals.

## Delay emergence

Reducing the need for and inappropriate use of antimicrobials can help delay emergence of AMR. In human health, extensive studies have been conducted in the United States, Europe, and other high-income settings on approaches to promote prudent antimicrobial prescribing – an approach known as antimicrobial stewardship.^19^ Success has been achieved when stewardship programmes include structural changes and behavioral modification techniques.^20,21,22^ Economic incentives for prescribing vary by country but can be particularly problematic when physicians or health care facilities rely on antimicrobial sales to fund operations. In many settings, persons obtain antimicrobials directly from pharmacies with no evaluation by or prescription from a physician.^23^ Additionally, substandard and counterfeit antimicrobials are widely available in Africa; substandard antimicrobials can promote AMR by containing levels of an agent sufficient to exert selective pressure for resistant organisms but insufficient levels to kill those organisms.^9^ There are limited studies about the effectiveness or essential components of antimicrobial stewardship programmes in Africa and other resource-limited settings, particularly involving the engagement of non-physician prescribers.^24^

In animals, antimicrobials are used for prophylaxis, treatment, and growth promotion, and it is reasonably assumed that most antimicrobial consumption occurs during food production, not through prescriptions to treat illness. Prudent use in these settings is a critical risk management approach to delaying emergence, but is challenging, because food producers have a strong economic incentive to use antimicrobials and weak incentive to use them prudently.^25,26^ The AU has made a high-level commitment to food safety, engaging partners from multiple sectors, including the industry. Africa CDC can leverage this commitment by making policymakers more aware of the link between food production, AMR emergence, and food-borne illness and by promoting prudent antimicrobial use in collaboration with animal health, agriculture, and other related authorities.

## Limit transmission

Transmission of AMR occurs frequently in health care facilities.^27^ Such transmission can have severe consequences, because pathogens in health care facilities are more likely to be multi-drug resistant. Hospitalised people are more susceptible to these severe illness, and these pathogens can also be spread outside of the hospital. The basic components of all programmes include: strong political commitment and dedicated resources for infection control, strict adherence to protocols for hand hygiene and for identification, isolation, and management of potentially infectious patients, adequate supplies and equipment for patient care, systems for infectious waste management, building design, and maintenance to reduce transmission, and continuous monitoring of process, outcome, and impact indicators.^28^ The West Africa Ebola response demonstrated that effective hospital-based infection prevention and control programmes require intensive support to initiate and sustain. Africa CDC’s framework, therefore focuses primarily on advocacy, policy, and guidance to mobilise sufficient resources for infection prevention and control.

In animal health, implementation of risk-based food safety systems – such as hazard analysis of critical control points, good handling practices, and good agricultural practices – can reduce the flow of AMR pathogens in food to humans, especially when combined with proper nutrition to reduce the need for antimicrobials.^29^

## Mitigate harm

Strong antimicrobial stewardship programmes promote adherence to clinical treatment guidelines, helping delay emergence of AMR and improve outcomes among patients already infected with AMR organisms. Diagnostic stewardship will also be essential to guide such treatment.^30^ Substandard diagnostic tests and laboratory supplies remain common problems across Africa and can lead to AMR when tests lead to unnecessary antibiotic treatment. Limiting their availability will be challenging, given the large number of health care delivery sites that may be using such products and the insufficient supply of quality diagnostic tests. A major ethical dilemma for Africa CDC’s framework will be to balance antibiotic access versus excess.^31^ Although antibiotic usage is excessive at a population level, many vulnerable groups lack access to effective antimicrobial treatment. Africa CDC’s framework focuses on promoting antimicrobial and diagnostic stewardship, while seeking ways to maintain access to life-saving diagnostics and treatments.

## Enablers of Africa Centres for Disease Control and Prevention’s framework

Laws and policies play a critical role in framing, enabling, and protecting public health. Africa CDC will leverage its stature and authority as a specialised agency of the AU to advocate for laws and policies to monitor, prevent, and mitigate AMR. Such policy initiatives, however, can only succeed with robust involvement of civil society. To date, engagement of civil society for AMR has been challenging, because the science can be complex to explain, the threat often characterised as distant, patients’ stories of illness and death often not told because of under-diagnosis and because they occur in marginalised groups, interventions not readily distilled into high-impact slogans, and public health agencies investing too little in civil society engagement.

To effectively control AMR, each country will need leadership and staff that have the education and skills to implement AMR control programmes in both human and animal health sectors. The most pronounced human resources gaps are likely in laboratory services – from bench microbiologists to managers – and in health care infection prevention and control. Africa CDC and partners are working together on a public health workforce strategy for the continent and mobilising resources to support hiring, training, and retaining of staff. By highlighting the urgency and severity of the AMR threat, Africa CDC will advocate for both donors and government ministries to allocate more funding to laboratory services and infection prevention and control personnel.

## Implementation of Africa Centres for Disease Control and Prevention’s framework

From 4–5 April 2018, Africa CDC convened AMR focal points from African countries and partners to review the Africa CDC AMR framework and prioritise activities for the next year. One major conclusion of the meeting was that Africa CDC should establish a formal structure to coordinate AMR activities within the AU, particularly with the AU’s animal health agency (the Inter-African Bureau for Animal Resources), across member states, and with multilateral and non-governmental partners in Africa. Participants proposed that this entity’s first task should be to transform the Africa CDC AMR framework into an AU-wide framework and ensure that it more fully addresses AMR issues in animal and environmental health. In addition to governance and coordination, participants proposed that Africa CDC focus activities in the next year on developing and harmonising tools to map and assess human and animal diagnostic laboratory capacity for AMR, developing guidelines for prudent antimicrobial use in humans and in animals that reflect what is currently known about AMR in Africa, and developing minimum standards for infection prevention and control in health care facilities to limit AMR transmission.

### Conclusion

Africa CDC’s framework for AMR is broad and aspirational, reflecting the urgency and complexity of this public health problem and the potential for Africa CDC as an institution to help galvanise action and provide guidance to member states. Progress in implementing this framework will require close coordination across and high-level commitment from member states and partners in Africa.

## References

[1] World Bank Drug-resistant infections: A threat to our economic future [homepage on the Internet]. Washington, DC; 2016 Available from: http://pubdocs.worldbank.org/en/689381474641399486/1701381-AMR-Lab-Report-Web.pdf

[2] Laxminarayan R, Duse A, Wattal C, et al Antibiotic resistance-the need for global solutions. Lancet Infect Dis. 2013;13:1057–1098. https://doi.org/10.1016/S1473-3099(13)70318-9 24252483 10.1016/S1473-3099(13)70318-9

[3] Ndihokubwayo JB, Yahaya AA, Desta AT, et al Antimicrobial resistance in the African region: Issues, challenges and actions proposed. Key determinants for health in the African region. African Health Monitor. 2013;16.

[4] World Health Organization Drug resistant tuberculosis is now at record levels [homepage on the Internet]. Geneva; 2010 Available from: http://www.who.int/mediacentre/news/releases/2010/drug_resistant_tb_20100318/en/

[5] Essack SY, Desta AT, Abotsi RE, Agoba EE Antimicrobial resistance in the WHO African region: Current status and roadmap for action. J Pub Health. 2016;39:8–13. https://doi.org/10.1093/pubmed/fdw015 10.1093/pubmed/fdw015 PMC5939661 26944074

[6] Le Doare K, Bielicki J, Heath PT, Sharland M Systematic review of antibiotic resistance rates among gram-negative bacteria in children with sepsis in resource-limited countries. J Pediatric Infect Dis Soc. 2015;4:11–20. https://doi.org/10.1093/jpids/piu014 26407352 10.1093/jpids/piu014

[7] Musicha P, Cornick JE, Bar-Zeev N, et al Trends in antimicrobial resistance in bloodstream infection isolates at a large urban hospital in Malawi (1998–2016): A surveillance study. Lancet Infect Dis. 2017;17(10):1042–1052. https://doi.org/10.1016/S1473–3099(17)30394-8 28818544 10.1016/S1473-3099(17)30394-8 PMC5610140

[8] Levy SB, Marshall B Antibacterial resistance worldwide: Causes, challenges and responses. Nat Med. 2004;10:S122–S129. https://doi.org/10.1038/nm1145 15577930 10.1038/nm1145

[9] Newton PN, Green MD, Fernández FM, Day NP, White NJ Counterfeit anti-infective drugs. Lancet Infect Dis. 2006;6:602–613. https://doi.org/10.1016/S1473-3099(06)70581-3 16931411 10.1016/S1473-3099(06)70581-3

[10] Okeke IN, Lamikanra A, Edelman R Socioeconomic and behavioral factors leading to acquired bacterial resistance to antibiotics in developing countries. Emerg Infect Dis. 1999;5(1):18–27. https://doi.org/10.3201/eid0501.990103 10081668 10.3201/eid0501.990103 PMC2627681

[11] O’Neill J Tackling drug-resistant infections globally: final report and recommendations [homepage on the Internet]. Wellcome Trust and HM Government c2018 [cited 2018 Nov 8]. Available from: https://amr-review.org/sites/default/files/160525_Final%20paper_with%20cover.pdf

[12] World Health Organization Global action plan on antimicrobial resistance [homepage on the Internet]. 2015 Available from: http://apps.who.int/iris/bitstream/10665/193736/1/9789241509763_eng.pdf 10.7196/samj.9644 26242647

[13] United Nations Political declaration of the high-level meeting of the General Assembly on antimicrobial resistance (A/RES/71/3) [homepage on the Internet]. Available from: https://digitallibrary.un.org/record/845917/files/A_RES_71_3-EN.pdf

[14] African Union Declaration on accelerating implementation of International Health Regulations in Africa [homepage on the Internet]. Doc. EX.CL/1026(XXXI). Available from: http://africacdc.org/resources/continental-commitments/continental-commitments/ihr-english/download

[15] Africa Centres for Disease Control and Prevention Framework for antimicrobial resistance, 2018–2023 [homepage on the Internet]. Available from: http://www.africacdc.org/resources/strategic-framework 10.4102/ajlm.v7i2.830 PMC6295971 30568906

[16] Nkengasong JN, Maiyegun O, Moeti M Establishing the Africa Centres for Disease Control and Prevention: Responding to Africa’s health threats. Lancet Glob Health. 2017;5:e246–e247. https://doi.org/10.1016/S2214-109X(17)30025-6 28108138 10.1016/S2214-109X(17)30025-6

[17] East Africa Public Health Laboratory Networking Project Report by Center for Disease Dynamics, Economics, and Policy [homepage on the Internet]. Washington, DC; 2016 Available from: https://www.cddep.org/sites/default/files/wb_report_32.pdf

[18] Nkengasong JN, Yao K, Onyebujoh P Laboratory medicine in low-income and middle-income countries: Progress and challenges. Lancet. 2018;391(10133):1873–1875. https://doi.org/10.1016/S0140-6736(18)30308-8 29550031 10.1016/S0140-6736(18)30308-8 PMC5962422

[19] Llor C, Bjerrum L Antimicrobial resistance: Risk associated with antibiotic overuse and initiatives to reduce the problem. Ther Adv Drug Saf. 2014;5(6):229–241. https://doi.org/10.1177/2042098614554919 25436105 10.1177/2042098614554919 PMC4232501

[20] Arnold SR, Straus SE Interventions to improve antibiotic prescribing practices in ambulatory care. Cochrane Database Syst Rev. 2005;(4):CD003539 https://doi.org/10.1002/14651858.CD003539.pub2 16235325 10.1002/14651858.CD003539.pub2 PMC7003679

[21] Davey P, Marwick CA, Scott CL, et al Interventions to improve antibiotic prescribing practices for hospital inpatients. Cochrane Database Syst Rev. 2017;(2):CD003543 https://doi.org/10.1002/14651858.CD003543.pub4 28178770 10.1002/14651858.CD003543.pub4 PMC6464541

[22] Hulscher ME, Grol RP, Van der Meer JW Antibiotic prescribing in hospitals: A social and behavioural scientific approach. Lancet Infect Dis. 2010;10:167–175. https://doi.org/10.1016/S1473-3099(10)70027-X 20185095 10.1016/S1473-3099(10)70027-X

[23] Morgan DJ, Okeke IN, Laxminarayan R, et al Non-prescription antimicrobial use worldwide: A systematic review. Lancet Infect Dis. 2011;11:692–701. https://doi.org/10.1016/S1473-3099(11)70054-8 21659004 10.1016/S1473-3099(11)70054-8 PMC3543997

[24] Cox JA, Vlieghe E, Mendelson M, et al Antibiotic stewardship in low-and middle-income countries: ‘Same, but different’? Clin Microbiol Infect. 2017;23(11):812–818. https://doi.org/10.1016/S1198-743X(17)30365-8 28712667 10.1016/j.cmi.2017.07.010

[25] World Animal Health Organization (OIE) Terrestrial animal health code – Chapter 6.7. In: Harmonization of national antimicrobial resistance surveillance and monitoring programmes [homepage on the Internet]. 2017 Available from: http://www.oie.int/fileadmin/Home/eng/Health_standards/tahc/current/chapitre_antibio_harmonisation.pdf

[26] Landers TF, Cohen B, Wittum TE, Larson EL A review of antibiotic use in food animals: Perspective, policy, and potential. Public Health Rep. 2012;127:4–22. https://doi.org/10.1177/003335491212700103 22298919 10.1177/003335491212700103 PMC3234384

[27] Weinstein RA Controlling antimicrobial resistance in hospitals: Infection control and use of antibiotics. Emerg Infect Dis. 2001;7:188–192. https://doi.org/10.3201/eid0702.010206 11294703 10.3201/eid0702.010206 PMC2631704

[28] World Health Organization Guidelines on core components of infection prevention and control [homepage on the Internet]. 2016 Available from: http://www.who.int/gpsc/ipc-components/en/27977095

[29] Food Animal Organization Impact of animal nutrition on animal welfare [homepage on the Internet]. 2011 Available from: www.fao.org/3/a-i3148e.pdf

[30] Morgan DJ, Malani P, Diekema DJ Diagnostic stewardship – Leveraging the laboratory to improve antimicrobial use. J Am Med Assoc. 2017;318:607–608. https://doi.org/10.1001/jama.2017.8531 10.1001/jama.2017.8531 28759678

[31] Das P, Horton R Antibiotics: Achieving the balance between access and excess. Lancet. 2016;387(10014):102–104. https://doi.org/10.1016/S0140-6736(15)00729-1 26603923 10.1016/S0140-6736(15)00729-1

